# Current trends in the surgical management of Dupuytren’s disease in Europe: an analysis of patient charts

**DOI:** 10.1007/s12570-012-0092-z

**Published:** 2012-03-06

**Authors:** Christopher Bainbridge, Lars B. Dahlin, Piotr P. Szczypa, Joseph C. Cappelleri, Daniel Guérin, Robert A. Gerber

**Affiliations:** 1 Pulvertaft Hand Centre, Royal Derby Hospital, Uttoxeter Road, Derby, UK; 2 Department of Clinical Sciences Malmö, Hand Surgery, Lund University, Malmö, Sweden; 3 Medical Affairs, Pfizer Ltd, Tadworth, Surrey UK; 4 Medicines Development Group, Pfizer Inc, Groton, CT USA; 5 A+A Healthcare Research, Lyon, France

**Keywords:** Dupuytren’s disease, Cord contracture, Fasciectomy, Fasciotomy, Percutaneous needle fasciotomy, Dermofasciectomy

## Abstract

**Introduction:**

Dupuytren’s disease (DD) causes progressive digital flexion contracture and is more common in men of European descent.

**Methods:**

Orthopaedic and plastic surgeons in 12 European countries (the Czech Republic, Denmark, Finland, France, Germany, Hungary, Italy, The Netherlands, Poland, Spain, Sweden and the UK) with >3 and <30 years experience reviewed the medical charts of five consecutive patients they had treated surgically for DD in 2008. Descriptive statistics are reported.

**Results:**

In total, 3,357 patient charts were reviewed. Mean (standard deviation) patient age was 61.9 (10.2) years; 81% were men. At the time of the procedure, 11% of patients were at Tubiana stage Ia (0–20° total flexion); 30%, stage Ib (21–45°); 34%, stage II (46–90°); 17%, stage III (91–135°); and 5%, stage IV (>135°). Percutaneous needle fasciotomy was performed in 10%, fasciotomy in 13%, fasciectomy in 69% and dermofasciectomy (DF) in 6% of patients. After surgery, fingers improved a mean of 1.9 Tubiana stages, and 54% of patients had no nodules or contracture. The rate of reported complications during the procedure was 4% overall (11% in patients undergoing DF). The most common postoperative complications reported were haematoma (8%), wound healing complications (6%) and pain (6%). No postoperative complications were reported in 77% of patients.

**Conclusions:**

In this European study of more than 3,000 patients with DD, most patients were diagnosed at Tubiana stage I or II, the majority received fasciectomy and more than half had no nodules or contracture remaining after surgery.

**Electronic supplementary material:**

The online version of this article (doi:10.1007/s12570-012-0092-z) contains supplementary material, which is available to authorized users.

## Introduction

Dupuytren’s disease (DD), a fibroproliferative condition of the hand causing progressive digital flexion contracture, most often affects older men of northern European descent [1] and is more common in patients with diabetes [2]. Estimates of prevalence range from less than 1% to greater than 50% depending on the population studied [3].

Treatment of Dupuytren’s contracture typically involves surgery. In order of aggressiveness, surgical procedures performed include percutaneous needle fasciotomy (PNF; also known as percutaneous needle aponeurotomy or needle fasciotomy), fasciotomy (subcutaneous or open), fasciectomy (also known as regional palmar fasciectomy or aponeurectomy), dermofasciectomy (DF) and amputation [4–7]. Fasciectomy has been reported as the most common surgical procedure performed for Dupuytren’s contracture in Europe. A recent analysis of hospital records in England found that more than 90% of inpatient and outpatient procedures for palmar fascial fibromatosis were classified as fasciectomy or revision of fasciectomy [8]. Using data collected from the French National Hospital Database, Maravic and Landais [9] found that 88% of procedures for DD were fasciectomies. In a retrospective analysis in Erlangen, Germany, Loos et al. [10] found that 95% of procedures for DD were limited fasciectomies and 5% were total fasciectomies.

In a recent systematic review, fasciectomy and fasciotomy were found to have similar efficacy, with mean improvement in degree of contracture ranging from 45% to 90% in various studies [4]. Recurrence occurred in approximately 40% of patients receiving fasciectomy and 60% of patients receiving fasciotomy, at a median time of about 4 years [4]. In another systematic review, measures of efficacy and recurrence were found to be inconsistent among studies, making it difficult to compare levels of efficacy between the procedure types. However, there was some evidence of a higher rate of recurrence after PNF than after open procedures [7].

While there have been many local studies of surgical interventions for Dupuytren’s contracture in Europe, to our knowledge there has been no large-scale study of surgical procedures for DD and their outcomes across Europe. Accordingly, the objective of this study was to assess, across Europe, the surgical treatment patterns for DD and outcomes in different stages of disease. This article and its companion article [11] concerning an associated surgeon survey, report general findings across all 12 countries.

## Methods

The study involved surgeons’ review of medical charts of patients for whom the surgeon had personally performed a surgical procedure for DD.

### Participating surgeons

Surgeons were recruited from 12 European countries: the Czech Republic, Denmark, Finland, France, Germany, Hungary, Italy, The Netherlands, Poland, Spain, Sweden and the UK. Details of recruitment and inclusion criteria for participating surgeons are described in the companion article [11].

Data collection took place between November 2009 and January 2010. Surgeons responded to a questionnaire via the Internet or during a face-to-face interview. Each surgeon reviewed the medical charts of approximately five patients they had personally treated with a surgical procedure for DD between September and December 2008, identified in sequential order in the surgeon’s records.

### Patients

To be included in the study, patients must have been diagnosed with DD and undergone a surgical procedure for the disease between September and December 2008. The surgical procedure must have been performed by an orthopaedic or plastic surgeon; hand surgeons were included in each of these groups. There were no exclusion criteria.

### Interviewers and online data collection

Before the initiation of the study, a central briefing meeting was conducted to review the study protocol and chart review instructions with all interviewers involved. When necessary, additional aid was provided to ensure the consistent collection of data, both in response to interviewers’ queries and through regular contact with the agencies overseeing the interview process.

### Surgical procedures

The following surgical procedures were identified in the questionnaire and defined as follows:

- Needle fasciotomy/aponeurotomy (referred to as PNF in this article): A small gauge hypodermic needle is inserted through a skin prick into the Dupuytren’s cord. The bevel of the needle is used as a blade to divide and release the contracting bands. This is a blind procedure under local anaesthetic. No tissue is removed during the procedure
- Fasciotomy: A single or multiple palmar/finger incisions are made above the Dupuytren’s cord and sharp dissection is performed to facilitate release. In this study, fasciotomy was defined as including the following:
  - Subcutaneous fasciotomy: The fascia is cut blindly with a small knife (a number 11 blade) via a stab wound skin incision. No tissue is removed during the procedure. This procedure is usually done under local anaesthetic.
  - Open fasciotomy: The overlying skin is opened exposing the cord. Under visual control, the surgeon is able to cut and release the Dupuytren’s cord, and the skin is closed without removing any tissue fascia. This procedure is usually done under local anaesthetic.
- Fasciectomy/aponeurectomy: This procedure excises the diseased fascia of the palm and/or digits. For the purposes of this research, fasciectomy includes the following terms: limited, local, partial, regional, selective, segmental, sub-total and total. The procedure requires general anaesthesia or nerve block. Rehabilitation and wound care are needed
- Dermofasciectomy: Removal of diseased fascia as well as diseased skin adjacent to the diseased fascia. This diseased skin is usually replaced with a skin graft
- Amputation of the affected digit/phalanx

### Questionnaire items

A questionnaire was used to elicit information from patient charts and included items on patient characteristics, referral history, diagnosis history, the procedure performed, outcome after the procedure and follow-up. Surgeons were instructed to report the Tubiana stage of Dupuytren’s contracture [12] in each affected finger by adding together the individual flexion deformities (deficiency extension) of the metacarpophalangeal (MCP), proximal interphalangeal (PIP) and distal interphalangeal (DIP) joints. The Tubiana classification scheme is widely used [7] to rate severity of Dupuytren’s contracture and was modified slightly for this investigation. Tubiana stages are described throughout this study as:

- Stage 0: no lesion, healthy
- Stage N: palmar or digital nodule without established flexion deformity
- Stage Ia: total flexion deformity between 0° and 20°
- Stage Ib: total flexion deformity between 21° and 45°
- Stage II: total flexion deformity between 45° and 90°
- Stage III: total flexion deformity between 91° and 135°
- Stage IV: total flexion deformity exceeding 135°.

The questionnaire was translated into the local language of each country by translation and fieldwork agencies. The translated questionnaires were checked by A+A Healthcare Research and local company affiliates. The text of the survey is provided as online supplementary material to this article.

### Quality assurance

The data collected was quality controlled before analysis by the A+A statistical group. Filters were put in place to exclude data values that were not logically possible (i.e. when the responding surgeon could not be contacted to correct the item). Answers outside the accepted range were queried with the physicians, and qualitative explanations were sought before the inclusion of the data in the dataset to be analysed. Data were checked for coherence at the level of the interview through coherence tests programmed in the questionnaire for both online and face-to-face interviews. Coherence was checked again before data processing; any data that did not conform to the coherence filters set were queried with the physician that had provided it through a recall process.

### Statistical analysis

Descriptive statistics were analysed and are reported as percentages and means with standard deviations (SD).

## Results

### Demographics of participants

A total of 687 surgeons participated in the study (Table 1). Of the responding surgeons, 579 (84%) were orthopaedic surgeons and 108 (16%) were plastic surgeons. Of the 687 participants, 383 (56%) were hand surgeons, including 339 orthopaedic surgeons and 44 plastic surgeons. Specific details of the number and types of surgeons interviewed from each country are provided in the companion article [11].

**Table 1:** Countries surveyed, number of respondents and number of patient charts reviewed

Country	Surgeon respondents	Patient cases reviewed
Czech Republic	40	200
Denmark	23	93
Finland	20	91
France	91	456
Germany	90	450
Hungary	50	250
Italy	90	450
The Netherlands	42	176
Poland	40	200
Spain	90	451
Sweden	18	90
UK	93	450
Total	687	3,357

The surgeons reviewed 3,357 patient charts, reflecting up to five patients per surgeon who had been treated surgically for Dupuytren’s contracture; most surgeons reviewed five patient charts. The greatest numbers of patients (approximately 450 per country) were from France, Germany, Italy, Spain and the UK. The fewest patients (approximately 90 per country) were from Denmark, Finland and Sweden.

Of the patients included, 2,734 (81%) were men. Where information about race could be queried, 99% (2,766 of 2,808) patients were Caucasian/white. The mean (SD) age of all patients was 61.9 (10.2) years; 1,229 (37%) were aged more than 65 years and 370 (11%) were aged less than 50 years. The following comorbidities and risk factors were reported among all patients: 1,412 (42%) smoked (more than five cigarettes per day), 663 (20%) had type 2 diabetes mellitus, 260 (8%) had type 1 diabetes mellitus, 578 (17%) consumed more than three alcoholic drinks per day, 320 (10%) had a personal history of Dupuytren’s contracture and 744 (22%) had a family history of Dupuytren’s contracture.

### Diagnosing and referring physicians

Of all patients, 1,654 (49%) were originally diagnosed by a general practitioner, 723 (22%) were diagnosed by the responding surgeon and 437 (13%) were diagnosed by another orthopaedic surgeon. The setting of the original diagnosis was a physician’s office for 1,576 (47%), an outpatient department for 911 (27%) and in a hospital for 557 (17%).

Among all patients, 1,836 (55%) were referred to the responding surgeon by a general practitioner, and 362 (11%) were referred by another orthopaedic surgeon. For 1,949 patients (58%), the reason for referral was the need for a procedure. For 267 patients (8%), the reason for the referral was for diagnosis or confirmation of diagnosis. A total of 856 patients (26%) were not referred (i.e. they came directly to the responding surgeon).

### Clinical profile at diagnosis

#### Symptoms and functional limitations

To the extent recorded by the responding surgeon, symptoms in all patients that originally led to a diagnosis of DD included finger flexion towards the palm (2,503; 75%), patient’s complaint about functionality (1,916; 57%), lump on the palm or fingers on physical examination (1,719; 51%), a positive tabletop test (1,204; 36%), patient’s complaint about appearance (953; 28%) and patient’s complaint about pain (564; 17%).

Lump on palm or fingers was a more common reason for diagnosis for patients with a lower Tubiana stage of disease, whereas finger flexion, positive tabletop test and complaint about functionality were more common reasons for diagnosis for patients with a higher Tubiana stage. The proportion of patients with pain recorded as a reason for diagnosis was similar (15% to 18%) for patients with all Tubiana stages. Surgeons reported that 1,896 (56%) of all patients had functional limitations at the time of diagnosis affecting leisure activities and that 1,919 (57%) had functional limitations affecting work activities.

#### Tubiana stage

**Fig. 1:**
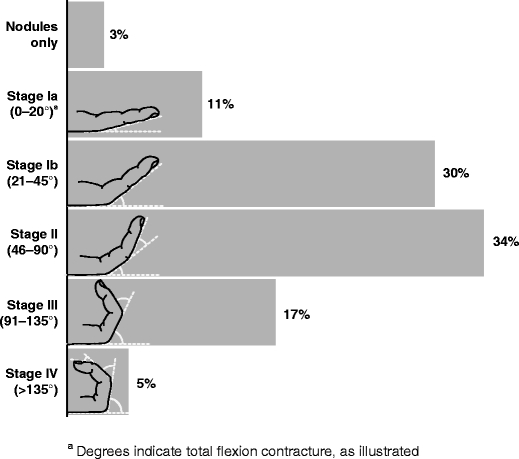
Tubiana stage at time of procedure

At the time of diagnosis, 155 (5%) of all patients were recorded as having nodules only, 429 (13%) were at Tubiana stage Ia (0–20° total flexion in the most severely affected finger), 1,017 (30%) were at stage Ib (21–45°), 1,066 (31%) were at stage II (46–90°), 502 (15%) were at stage III (91–135°) and 155 (5%) were at stage IV (more than 135°) (see Fig. 1 showing stage at time of procedure). Older patients and men were more often diagnosed with higher stages of disease.

#### Number of hands, fingers and joints affected

Of all patients, 2,951 (88%) were diagnosed with DD in only one hand. Of 2,826 right-handed patients, 1,767 (63%) were diagnosed with Dupuytren’s in the right hand only; of 334 left-handed patients, 237 (71%) were diagnosed in the left hand only. Of 43 patients identified as ambidextrous, 16 (37%) were diagnosed in the right hand only; 18 (42%) were diagnosed in the left hand only, and nine (21%) were diagnosed in both hands.

**Fig. 2:**
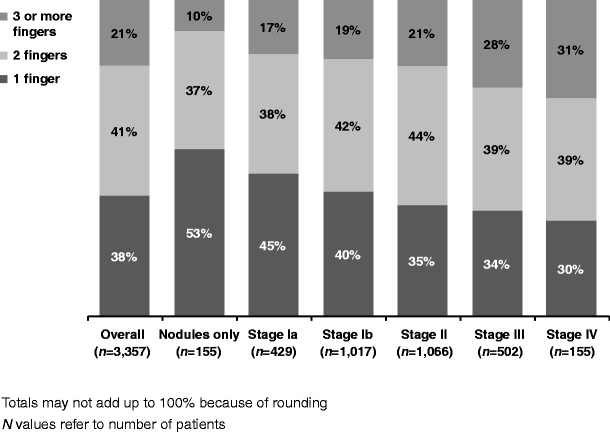
Number of fingers affected by stage of disease at time of diagnosis

Of all patients, 1,280 (38%) were diagnosed with Dupuytren’s in only one finger, 1,381 (41%) in two fingers and 696 (21%) in three or more fingers (where fingers included the thumb). Patients diagnosed with a higher Tubiana stage had more fingers involved more often (Fig. 2).

Of all patients, 228 (7%) were affected in zero joints (had nodules only), 577 (17%) were affected in one joint, 978 (29%) were affected in two joints, 432 (13%) were affected in three joints and 569 (17%) were affected in four joints. The remaining 573 (17%) patients were affected in five or more joints.

### Procedure performed

#### Tubiana stage at time of procedure

A mean (SD) of 29.9 (46.4) months elapsed between initial diagnosis and procedure. Patients’ Tubiana stage at the time of procedure was similar to the stage at the time of diagnosis: 106 (3%) of all patients had nodules only, 366 (11%) were at Tubiana stage Ia, 999 (30%) were at stage Ib, 1136 (34%) were at stage II, 567 (17%) were at stage III and 164 (5%) were at stage IV. The distribution of Tubiana stages was similar for patients treated by orthopaedic, plastic and hand surgeons.

#### Procedures performed

**Fig. 3:**
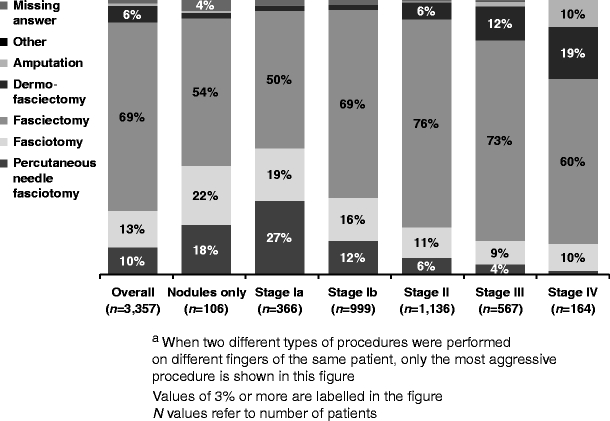
Most aggressive procedure performed^a^ by stage of disease at time of procedure

The most aggressive surgical procedure performed for each patient is shown in Fig. 3. Of 3,357 procedures performed, 329 (10%) were PNFs, 446 (13%) were fasciotomies, 2,311 (69%) were fasciectomies, 200 (6%) were DFs and 34 (1%) were amputations. More aggressive procedures were more often performed for patients with higher stage of disease. The distribution of procedure types performed was similar across surgical specialties and similar across patients regardless of risk factors or comorbidities.

#### Number of hands, fingers and joints operated

Of all patients, 3,249 (97%) were operated on only one hand. More patients had only one finger (1,381; 41%) or on two fingers (1,459; 43%) operated than on three or more fingers (517; 15%). Patients with a higher stage of disease were more likely to have more fingers operated, and the number of fingers operated did not differ appreciably by surgeon specialty.

Fingers operated closely matched the fingers diagnosed: 2,852 (85%) of all patients were operated on the same fingers as those diagnosed, 373 (11%) on fewer fingers than at diagnosis, 89 (3%) on different fingers than at diagnosis and 43 (1%) on more fingers than at diagnosis. Of all patients, 1,353 (62%) were operated on a small finger, 1,496 (68%) on a ring finger, 660 (30%) on a middle finger, 223 (10%) on an index finger and 86 (4%) on a thumb.

Overall, a mean (SD) of 2.9 (1.9) joints per patient and 1.7 (0.7) joints per finger were operated. Of 5,984 fingers operated, the MCP joint was operated in 4,814 (80%), the PIP joint in 3,958 (66%) and the DIP joint in 1,202 (20%).

#### History of previous surgery

Of all patients, 216 (6%) had already received surgery on the same finger that a procedure was reported for in this study; the previous surgery had taken place a mean of 54.7 (SD, 38.4) months earlier. The proportion who had already received surgery on the same finger was greater (38 of 200 patients, 19%) among patients who received DF in this study.

Of 420 fingers that were reoperated, 216 (51%) received fasciectomy in this study. Fingers receiving the same procedure previously performed included 19 (68%) of 28 receiving PNF in this study, 13 (26%) of 50 receiving fasciotomy, 157 (55%) of 284 receiving fasciectomy and 4 (7%) of those 54 receiving DF.

#### Site of operation and operating time

**Fig. 4:**
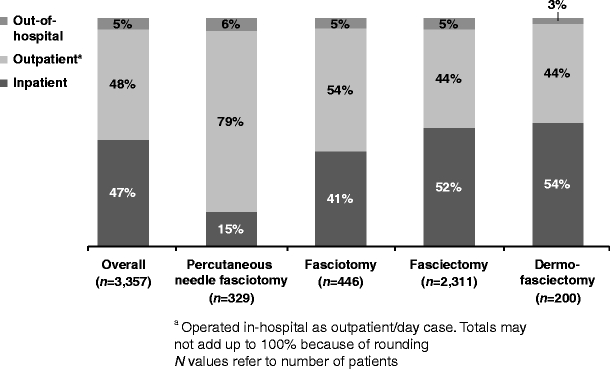
Hospitalization patterns for patients receiving each procedure type

More aggressive procedures were performed more often on an inpatient basis (Fig. 4). Mean operation time was 61.2 (SD, 33.1) min. Operation time was less than 30 min for 260 (8%), 30 to 60 min for 2,049 (61%) and more than 1 h for 1,036 (31%) of the 3,345 procedures for which this information was available. Mean operation time was longer for more aggressive procedures: The mean (SD) time to perform the procedure was 38.7 (26.3) min for PNF, 53.3 (33.8) min for fasciotomy, 63.5 (30.2) min for fasciectomy and 89.7 (42.8) min for DF.

As shown in Fig. 4, most patients were not admitted as inpatients for their surgery. For patients who were admitted to the hospital, the mean (SD) number of nights spent in the hospital was 2.3 (1.6) for those receiving PNF, 2.0 (1.4) for fasciotomy, 2.3 (1.5) for fasciectomy and 2.8 (2.1) for DF.

#### Bandaging and splinting

Immediately after the procedure, a bulky bandage was applied for 1,627 (48%) of all patients, a light dressing for 1,041 (31%), a plaster slab for 612 (18%) and a thermoplastic splint for 396 (12%); a single patient might receive more than one dressing or splint. The postoperative dressing or splint used varied by procedure type. Overall, 1,360 (41%) of all patients were given a night splint, which was used for a mean (SD) of 32.2 (38.1) nights. Splints were used for a longer period of time following fasciectomy and DF than following PNF and fasciotomy.

### Outcome of procedure

#### Tubiana stage after procedure

Surgeons reported the best (optimal) result recorded during the year after surgery. A mean of 2.9 (SD, 1.8) months elapsed after surgery before the optimal result for the patient was obtained. This was less than 3 months in 1,677 (50%) of all patients, 3 to 6 months in 1,588 (47%) and more than 6 months in 86 (3%). Mean (SD) time to obtain the optimal result was greater for more aggressive procedures: 2.2 (1.5) months after PNF, 2.8 (1.8) months after fasciotomy, 2.9 (1.7) months after fasciectomy and 3.5 (2.2) months after DF.

**Fig. 5:**
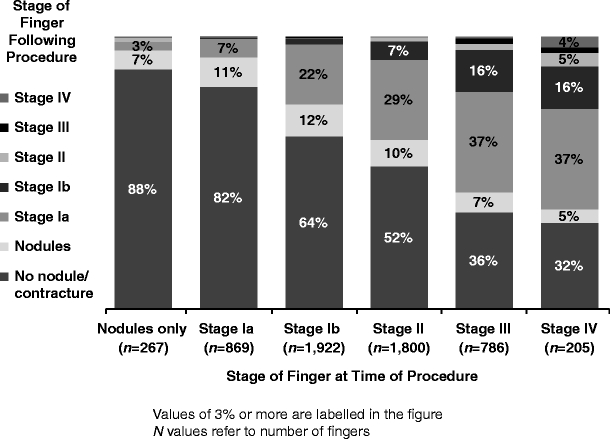
Outcome Tubiana stage after procedure

Figure 5 illustrates the Tubiana stage of each finger before and after the surgical procedure. The optimal Tubiana stage achieved after surgery was lower than the presurgery stage for 3,196 (96%) of all patients; 96 (3%) remained at the same stage and 22 (1%) had a more severe stage after surgery. The optimal result achieved after surgery was ‘no nodules/no contracture’ for 1,800 (54%) of all patients, nodules only for 345 (10%), stage Ia for 878 (26%), stage Ib for 237 (7%), stage II for 42 (1%) and stage III or IV for 29 (1%). At the time of the optimal result, 1,800 (54%) of all patients had no fingers affected by Dupuytren’s, 816 (25%) had one affected finger, 582 (17%) had two affected fingers and 133 (4%) had three or more affected fingers. The mean (SD) number of stages of improvement after surgery over all fingers was 1.9 (1.1); this was 1.5 (0.9) after PNF, 1.7 (1.1) after fasciotomy, 1.9 (1.1) after fasciectomy and 2.0 (1.2) after DF.

#### Complications and adverse events

Of all patients, 3,230 (96%) experienced no complications during the procedure (Table 2). Complications were most frequently reported in patients receiving DF, 25 (12%) of whom experienced a complication, including 10 (5%) who experienced nerve injury.

**Table 2:** Complications occurring during procedures

	Total^a^ (*n* = 3,357)	Percutaneous needle fasciotomy (*n* = 329)	Fasciotomy (*n* = 446)	Fasciectomy (*n* = 2,311)	Dermofasciectomy (*n* = 200)
None	3,230 (96%)	323 (98%)	438 (98%)	2,223 (96%)	179 (90%)
Artery injury	32 (1%)	3 (1%)	1 (0.2%)	22 (1%)	5 (3%)
Nerve injury	67 (2%)	1 (0.3%)	2 (0.5%)	51 (2%)	10 (5%)
Tendon injury	8 (0.2%)	1 (0.3%)	0	4 (0.2%)	3 (2%)
Volar plate injury	27 (1%)	2 (1%)	4 (1%)	14 (1%)	7 (4%)

^a^Total includes patients for whom procedure type was not known (*n* = 37) or who underwent amputation (*n* = 34)

Of all patients, 2,571 (77%) reported no postoperative complications (Table 3). The most common postoperative complications reported in all patients were haematoma (283; 8%), wound healing complications or delayed healing (207; 6%) and pain (213; 6%). Postoperative complications occurred more frequently in patients undergoing more aggressive procedures.

**Table 3:** Complications reported after procedures

	Total^a^ (*n* = 3,357)	Percutaneous needle fasciotomy (*n* = 329)	Fasciotomy (*n* = 446)	Fasciectomy (*n* = 2,311)	Dermofasciectomy (*n* = 200)
None	2,571 (77%)	308 (94%)	363 (81%)	1,720 (74%)	125 (63%)
Infection	73 (2%)	2 (<1%)	9 (2%)	54 (2%)	8 (4%)
Haematoma	283 (8%)	9 (3%)	21 (5%)	226 (10%)	20 (10%)
CRPS^b^	18 (<1%)	0	0	17 (<1%)	1 (<1%)
Inflammation	129 (4%)	2 (<1%)	24 (5%)	76 (3%)	23 (12%)
Finger required amputation	7 (<1%)	0	0	4 (<1%)	1 (<1%)
Abnormal sensitive reactions	81 (2%)	0	5 (1%)	63 (3%)	11 (6%)
Necrosis	85 (3%)	0	2 (<1%)	67 (3%)	16 (8%)
Pain	213 (6%)	7 (2%)	32 (7%)	142 (6%)	27 (14%)
Carpal tunnel syndrome/					
ulnar nerve compression	5 (<1%)	0	2 (<1%)	3 (<1%)	0
Wound healing complications/delayed healing	207 (6%)	2 (<1%)	10 (2%)	155 (7%)	38 (19%)
Other	7 (<1%)	0	1 (<1%)	5 (<1%)	0
Do not know	29 (1%)	3 (1%)	2 (<1%)	22 (1%)	1 (<1%)

^a^Total includes patients for whom procedure type was not known (*n* = 37) or who underwent amputation (*n* = 34)

^b^Complex regional pain syndrome or reflex sympathetic dystrophy or algodystrophy

Among 329 patients receiving PNF, there were no complications leading to readmission. Among 445 patients receiving fasciotomy, there were two complications leading to readmission, both of which involved infection. Among 2,308 patients receiving fasciectomy, there were 26 complications leading to readmission, of which nine involved haematoma, seven involved infection and four required amputation. Among 200 patients receiving DF, there were 11 complications leading to readmission. These complications involved pain (six patients), abnormal sensitive reactions (two patients), infection (two patients) and haematoma (two patients), and one patient required amputation.

#### Follow-up care

During the year after the procedure was performed, patients had a mean of 3.8 (SD, 2.1) visits with the responding surgeon. Following the procedure, 1,335 (40%) of all patients remained in the care of the responding surgeon only. This proportion varied somewhat for hand surgeons and nonhand surgeons: 705 (38%) of 1,875 patients managed by hand specialists were cared for by the surgeon only, compared with 545 (45%) of 1,216 patients managed by nonhand surgeons. Of the remaining patients, 1,271 (38%) were cared for by a physiotherapist or occupational therapist, 486 (14%) by a general practitioner and 284 (8%) by another surgeon.

#### Final condition after surgery and recurrence

At the final evaluation after surgery, which took place 3 months to 1 year after the procedure, patients’ Tubiana stage was very similar to the optimal stage achieved after surgery. At the final evaluation, disease stage was no nodules/no contracture for 1,718 (52%) of all patients, nodules only for 330 (10%), stage Ia for 881 (26%), stage Ib for 233 (7%), stage II for 39 (1%) and stage III or IV for 16 (0.5%).

Regarding patients’ hand function, function in work activities was improved for 1,776 (53%) of all patients, was not improved in 85 (3%) and was unknown in 58 (2%). The remaining patients had no record of work limitations in their charts. Function in leisure activities was improved for 1,725 (51%) of all patients, was not improved in 81 (2%) and was unknown in 90 (3%). There was little change in employment status after surgery.

During the year after surgery, 3,275 (98%) of all patients had no further surgical procedures for DD. Of all patients, 27 (1%) had a surgical procedure because of recurrence on the same finger or joint, 23 (1%) had a previously planned procedure on another joint and 32 (1%) had a procedure on another joint because of disease progression. A mean of 8.4 (SD, 4.4) months elapsed between the initial and later procedures. The rate of reoperation on the same finger or joint was the highest for patients who initially received DF; this occurred in 13 (7%) of 200 patients.

When asked about future plans, 3,013 (90%) of all patients said they did not plan treatment in the next 12 months. Surgeons planned to reoperate on a total of 613 fingers out of 5,961 fingers originally operated (10% of operated fingers) belonging to 334 (10%) of all patients.

#### Surgeons’ assessment of outcome

Surgeons reported that they assessed the effectiveness of the surgical procedures performed by measuring postoperative extension or flexion for 1,881 (56%) of all patients, assessing functional ability after surgery for 1,511 (45%) or by conducting a tabletop test for 557 (17%). Surgeons described the clinical outcome of the procedure as positive for 3,296 (98%) of all patients.

## Discussion

In this survey, the majority of physicians who conducted surgery for DD were orthopaedic surgeons, of whom more than half identified themselves as hand surgeons. In some countries, a number of surgeries were also performed by plastic surgeons, some of whom were hand surgeons as well. As would be expected, approximately half of all patients were referred to the surgeon by their general practitioner; a quarter of all patients visited the surgeons on their own.

The surgeons’ review of their patients’ charts indicated that the majority of patients affected with DD were male, which is not unexpected given the historical reports of this disease [3, 13]. Among the comorbidities observed in this study, diabetes is notable. DD is common in patients with type 1 and type 2 diabetes but is generally moderate in presentation and rarely requires surgery [2]. DD is also common in patients with impaired glucose tolerance [2]. Almost 30% of all patients in this study had type 1 or more often type 2 diabetes; these patients had DD severe enough to warrant surgery.

The relatively large proportion of patients reporting pain in this study was surprising, given that DD is not usually associated with pain [12]. However, similar to patients described by Viljanto [14], it is possible that some patients’ pain reported in this study was related to a comorbid pathology and not to DD itself.

Most patients in this study reported only one hand affected by DD in contrast with previous literature reporting more common bilateral involvement [12]. The reason for this difference is unclear; it is possible that surgeons may not have examined the other hand if no difficulty was reported by the patient or that the second hand was examined but mild DD was not noted in the patient’s chart. Additionally, contracture may have developed in the second hand at some time after the initial diagnosis; this would not have been captured in this chart review. Also in contrast to previous literature, more procedures involving DIP joints were noted in this study than expected. Involvement of the DIP joint is less commonly reported in the literature as compared with the MCP and PIP joints [12].

Based on this patient chart review, it was noted that the Tubiana stage of patients was similar at diagnosis and at the time of the procedure; approximately 60% of patients were at stage Ia/Ib, even though patients spent a substantial amount of time on the waiting list for surgery [11]. This is consistent with what is known about slow progression of the disease.

Improvement to a lower Tubiana stage was noted in most patients after surgery. This improvement is consistent with previous reports of successful surgical correction for Dupuytren’s contracture in European patients [4, 5, 7]. As expected, more aggressive procedures were performed for patients at higher Tubiana stages, with the most common procedure being fasciectomy. This is in line with previous reports on treatments for DD [4].

Surgeons reported that most patients experienced no complications during or after surgery. Complications were more common among patients receiving DF, most likely owing to the severity of disease in these patients and the more complicated surgical procedure. It is possible that some complications reported in this study were related to co-occurring hand disorders such as carpal tunnel syndrome or ulnar nerve compression.

Very few patients in this study were reoperated within 1 year because of a recurrence of contracture in the same finger/joint. A wide range of recurrence rates is reported in the literature, and the low rate in this study falls in this range [4, 7, 15]. The mechanisms and predictors of recurrence in DD are obscure, but hypotheses exist [16–18]. In evaluating the low reported rate of recurrence in this study, it must be emphasized that recurrence was evaluated only up to 1 year after surgery, whereas recurrence has been reported in the literature to occur most often between 3.3 and 4.4 years after surgery [4]. Moreover, reporting recurrence was not specifically requested in this study, assessment and definition of recurrence is variable [7] and patients may not have returned to the surgeon unless functional limitations occurred. Therefore, some cases of recurrence that did not lead to reoperation within the year of follow-up may have been missed.

To our knowledge, this is the first large survey of DD conducted in Europe. The large sample size allowed for efficient estimates of patient characteristics and types of procedures used across much of Europe. This study has limitations. First, this was a retrospective review of patient charts using a prespecified questionnaire; therefore, surgeons were reporting information captured in the chart at the time of treatment. Not all patient charts contained the information on all areas queried, such as hand function limitations in work and leisure activities. Second, because of the nature of surveys, inconsistencies could have been present across countries, and it is difficult to identify or account for them. Third, the questionnaire used in this study did not ask detailed questions about the techniques employed in each surgery (e.g. the use of Z-plasty procedures and local flaps); therefore, differences in outcomes related to specific surgical techniques would not have been captured.

An additional limitation of this study is that the chart review did not capture costs of patient care, which include direct (e.g. surgery, rehabilitation) and indirect (e.g. sick leave) costs [19, 20]. Direct costs include time in surgery, which in this study ranged from almost 40 min on average for PNF to 90 min for DF. Direct costs also include time for preoperative procedures, including preparation of anaesthesia and time spent in postoperative care. Other direct costs include hospitalization; patients admitted for surgery in this study spent about two to three nights in the hospital. Indirect costs, such as sick leave from work, can be even more substantial than direct costs [19, 21].

It will be interesting to observe how the treatment patterns observed in this study change as new options for managing DD become available. We advise repeating this study in 5 years to assess changes in surgical practice, the proportion of nonsurgical procedures being performed and consequent effects on recurrence.

## Electronic supplementary material

Below is the link to the electronic supplementary material.

ESM 1 (PDF 476 kb)
