# Supplementary material for: Current trends in the surgical management of Dupuytren’s disease in Europe: an analysis of patient charts
Source: Eur Orthop Traumatol. 2012 Mar 6;3(1):31–41. doi: 10.1007/s12570-012-0092-z (PMC3338000; doi:10.1007/s12570-012-0092-z)
Supplement: Supplementary file 1 — (PDF 476 kb) [file 12570_2012_92_MOESM1_ESM.pdf]

Supplementary material

Article title: **Current trends in the surgical management of Dupuytren's disease in Europe: an analysis of patient charts**

Journal: EOTR

Christopher Bainbridge • Lars B. Dahlin • Piotr P. Szczypa • Joseph C. Cappelleri • Daniel Guérin • Robert A. Gerber

C. Bainbridge (✉)

Pulvertaft Hand Centre, Royal Derby Hospital,

Kings Treatment Centre (KTC) Level 2, Uttoxeter Road,

Derby DE22 3NE, UK

e-mail: chris@hand-surgery.co.uk

Tel: +44 01332 554700

Fax: +44 01332 554800

L. B. Dahlin

Department of Clinical Sciences - Hand Surgery, Lund University, Skåne University Hospital Malmö, Malmö, Sweden

P. P. Szczypa

Medical Affairs, Pfizer Ltd., Tadworth, Surrey, UK

J. C. Cappelleri • R. A. Gerber

Medicines Development Group, Pfizer Inc, Groton, CT, USA

D. Guérin

A+A Healthcare Research, Lyon, France

Questionnaire items

PATIENT'S CHARACTERISTICS

Q1. Date of Birth: |__|__|__|__| YYYY

Q2. Gender (Responses: Male/Female)

Q3. Ethnicity (Responses: [African/Black]/Asian/[Caucasian/White]; not collected in France and Sweden)

Q4. Type of health coverage under which patient is being treated for their Dupuytren's contracture (Responses: [National/Public health insurance]/Private insurance/[No coverage / 100% out of pocket]/Unknown; could indicate as many as appropriate)

Q5. Did the procedure take place in: (Responses: Private hospital/Public hospital)

Q6. How would you describe the patient in terms of his/her dominant hand? (Responses: Left-handed/Right-handed/Ambidextrous/Unknown)

Q7. How did you classify your patient's profession/employment status at the time of surgery conducted between September to Dec 2008? (Responses: Unemployed/Retired/[Temporary disability/sick leave]/Permanent disability/[Non manual labor (Office based)]/Unskilled manual labor/[Skilled manual labor (e.g. craftman)]/Unknown)

Q8. Has the patient's profession/employment status changed since their surgery for Dupuytren's contracture? (Responses: Yes/No/Don't know)

Q8b. If yes: How do you classify your patient's profession/employment status after the surgery conducted between September to Dec 2008? (Responses: Unemployed/Retired/[Temporary disability/sick leave]/Permanent disability/[Non manual labor (Office based)]/Unskilled manual labor/[Skilled manual labor (e.g. craftman)]/Unknown)

Q10. Does the patient suffer from any co-morbidities or risk factors? (Tick as many as appropriate)

1 ❑ Type 1 Diabetes / IDDM

2 ❑ Type 2 Diabetes / NIDDM

3 ❑ Consumes more than 3 alcoholic drinks per day

4 ❑ Smokes more than 5 cigarettes per day

5 ❑ Epilepsy

6 ❑ Non-epileptic seizure disorder

7 ❑ Knuckle pads (Garrod's pads)

8 ❑ Ledderhose

9 ❑ Peyronie’s disease

10 ❑ Past history of trauma on left hand

11 ❑ Past history of trauma on right hand

12❑ Previous history of Dupuytren’s contracture

13❑ Family history of Dupuytren’s contracture

14❑ Other comorbidity or risk factor that could exacerbate Dupuytren’s contracture: specify _______________

DIAGNOSIS HISTORY

Q11. To the best of your knowledge, when was this patient first diagnosed with Dupuytren’s contracture?

|__|__| month (MM) / |__|__|__|__| year (YYYY) (or Unknown)

Q12. Who performed the initial diagnosis? (Responses: Yourself/GP/Orthopedic surgeon/Hand surgeon/Plastic surgeon/Rheumatologist/)

Q13. Where did the initial diagnosis take place? (Responses: In hospital/In outpatient department/In physician office outside of hospital/Other: specify/Don’t know)

Q14. What were the signs/symptoms for the diagnosis of a Dupuytren’s contracture (tick all that apply)?

1 ❑ Lump on the palm or fingers after physical examination

2 ❑ Fingers flexion towards the palm

3 ❑ Positive table top test

4 ❑ Patient’s complaint about appearance.

5 ❑ Patient’s complaint about functionality

6 ❑ Patient’s complaint about pain

7 ❑ Other patient complaints, specify___________________

8 ❑ Other, specify___________________

Q15-17. In the following table, please tick all the fingers and joints affected **at the time of initial diagnosis**. Then, for each affected finger **at the time of initial diagnosis**, please specify the stage of Dupuytren’s contracture at that time using the Tubiana’s classification of disease.

In the Tubiana scoring classification of Dupuytren’s disease severity, the total deformities are measured by adding together the individual flexion deformities (deficiency extension) of the MCP, PIP and DIP joints.

Stage 0 = no lesion, healthy

Stage N = palmar or digital nodule without established flexion deformity

Stage 1a = total flexion deformity between 0° and 20°

Stage 1b = total flexion deformity between 20° and 45°

Stage 2 = total flexion deformity between 45° and 90°

Stage 3 = total flexion deformity between 90° and 135°

Stage 4 = total flexion deformity exceeding 135°

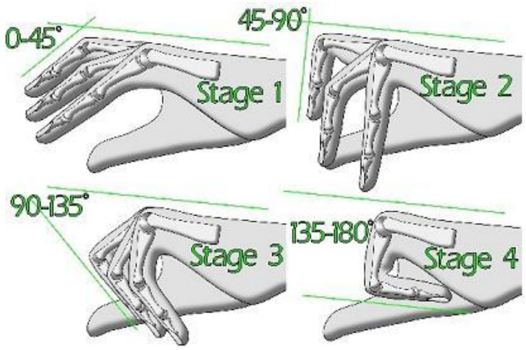

	**LEFT HAND**					**RIGHT HAND**				
	Thumb	Index	Middle	Ring	Baby	Baby	Ring	Middle	Index	Thumb
Q15. Please specify which fingers were affected at time of	1❑	2❑	3❑	4❑	5❑	6❑	7❑	8❑	9❑	10❑

initial diagnosis (tick as many as appropriate)										
Q16. Please specify joints affected										
DIP		1❑	1❑	1❑	1❑	1❑	1❑	1❑	1❑	
PIP	2❑	2❑	2❑	2❑	2❑	2❑	2❑	2❑	2❑	2❑
MCP	3❑	3❑	3❑	3❑	3❑	3❑	3❑	3❑	3❑	3❑
Q17. Stage of Dupuytren's Disease at time of initial diagnosis										

(total finger flexion adding up all the joints affected)										
N (Nodules)	1❑	1❑	1❑	1❑	1❑	1❑	1❑	1❑	1❑	1❑
Ia (< 20°)	2❑	2❑	2❑	2❑	2❑	2❑	2❑	2❑	2❑	2❑
Ib (20 – 45°)	3❑	3❑	3❑	3❑	3❑	3❑	3❑	3❑	3❑	3❑
II (45° - 90°)	4❑	4❑	4❑	4❑	4❑	4❑	4❑	4❑	4❑	4❑
III (90° - 135°)	5❑	5❑	5❑	5❑	5❑	5❑	5❑	5❑	5❑	5❑
IV (>135°)	6❑	6❑	6❑	6❑	6❑	6❑	6❑	6❑	6❑	6❑

Q18. *Only if fingers at stage N (nodule) at time of diagnosis (Q17=1),* when did the first flexion occur?

|__|__| month (MM) / |__|__|__|__| year (YYYY)

1❑ Unknown

2❑ No known progression to flexion

Q19. Do you know if the Dupuytren's contracture was coded under a Diagnosis Related Group (DRG) (or equivalent)? (Responses: Yes/No/Don't know)

If yes, please provide the DRG Code (or equivalent)? ___________________________

Q20. At time of diagnosis, did the patient report any of the following hand function limitations in his/her daily life?

	Q20a. Tick if functional limitation existed at diagnosis:	Q20b. *If ticked in Q20a*:<br>Did the patient complain of these functional limitations?
Work activities in general	1❑	1❑ Yes 2❑ No 3❑ Don't know
Leisure activities in general	2❑	1❑ Yes 2❑ No 3❑ Don't know
Washing or grooming	3❑	1❑ Yes 2❑ No 3❑ Don't know
Shaking hands	4❑	1❑ Yes 2❑ No 3❑ Don't know
Placing hand in pocket	5❑	1❑ Yes 2❑ No 3❑ Don't know
Putting on a glove	6❑	1❑ Yes 2❑ No 3❑ Don't know
Difficulty using fingers to grasp objects	7❑	1❑ Yes 2❑ No 3❑ Don't know
Applauding	8❑	1❑ Yes 2❑ No 3❑ Don't know
Using a computer or typing	9❑	1❑ Yes 2❑ No 3❑ Don't know

Recreational or sport activities such as golf or tennis	10❑	1❑ Yes 2❑ No 3❑ Don't know
Baking	11❑	1❑ Yes 2❑ No 3❑ Don't know
Playing a musical instrument	12❑	1❑ Yes 2❑ No 3❑ Don't know
Gardening	13❑	1❑ Yes 2❑ No 3❑ Don't know
Other: Specify _______________	14❑	1❑ Yes 2❑ No 3❑ Don't know
Other: Specify _______________	15❑	1❑ Yes 2❑ No 3❑ Don't know
The patient reported no impact on their life	16❑	

REFERRAL HISTORY

Q21. Was the patient referred to you by another physician? (Responses: Yes/No)

Q22. (If Q21 is Yes) What was the specialty of the physician who referred this patient to you?

1 ❑ General Practitioner / Primary Care Practitioner

2 ❑ Internal Medicine Physician

3 ❑ Geriatrician

4 ❑ Rheumatologist

5 ❑ Orthopedic Surgeon

6 ❑ Orthopedic surgeon specializing in hand surgery

7 ❑ Plastic Surgeon

8 ❑ Plastic Surgeon specializing in hand surgery

9 ❑ Other (please specify) _________________________________

Q23. (If Q21 is Yes) When was this patient referred to you?

|__|__| month (MM) / |__|__|__|__| year (YYYY)

Q24. (If Q21 is Yes) What was the principal reason for referring this patient to you?

1 ❑ Patient needed procedure

2 ❑ Patient needed medical treatment

3 ❑ Diagnosis was required or needed to be confirmed

4 ❑ Physician seeing the patient did not feel comfortable prescribing/making treatment decisions

5 ❑ Other (please specify): …………………………

PROCEDURE PERFORMED BETWEEN SEPTEMBER AND DECEMBER 2008

In this section of the questionnaire, we would like to focus on the procedure performed between September and December 2008

Q25a. Date of this surgery? (Responses: September 2008/October 2008/November 2008/December 2008)

Q25b.Where was the procedure performed?

1 ❑ In hospital as in-patient (go to Q26)

2 ❑ In hospital as out-patient (go to Q27).

3 ❑ In hospital as out-patient day case (go to Q27).

4 ❑ Out of hospital

Q26. *If the patient was an in-patient (Q25b=1)*, how many nights did the patient spend in the hospital?

|____| nights

❑ Don’t know?

Q27. *If the patient was treated as an out-patient or in the day case setting (Q25b=2 or 3)*, how many hours did the patient spend in the hospital / out-patient ward?

|____| hours

❑ Don’t know?

Q28. Was the Dupuytren’s Contracture procedure coded under ICD 10 / *OPCS (in the UK)* or an equivalent system?

❑ Yes ❑No

If yes, please provide the Code ___________________________

Q29. Please specify which fingers were operated on (tick as many as appropriate)

	**LEFT HAND**					**RIGHT HAND**				
	Thumb	Index	Middle	Ring	Baby	Baby	Ring	Middle	Index	Thumb
Q29	1❑	2❑	3❑	4❑	5❑	6❑	7❑	8❑	9❑	10❑

For each operated finger please specify:

Q30. Joints treated by the procedure

	**LEFT HAND**					**RIGHT HAND**				
	Thumb	Index	Middle	Ring	Baby	Baby	Ring	Middle	Index	Thumb
DIP		1❑	1❑	1❑	1❑	1❑	1❑	1❑	1❑	
PIP	2❑	2❑	2❑	2❑	2❑	2❑	2❑	2❑	2❑	2❑

MCP	3❑	3❑	3❑	3❑	3❑	3❑	3❑	3❑	3❑	3❑

Q31. Stage of Dupuytren's contracture at time of the procedure (total finger flexion adding up all the joints affected)

	LEFT HAND					RIGHT HAND				
	Thumb	Index	Middle	Ring	Baby	Baby	Ring	Middle	Index	Thumb
N (Nodules)	1❑	1❑	1❑	1❑	1❑	1❑	1❑	1❑	1❑	1❑
Ia (< 20°)	2❑	2❑	2❑	2❑	2❑	2❑	2❑	2❑	2❑	2❑
Ib (20° – 45°	3❑	3❑	3❑	3❑	3❑	3❑	3❑	3❑	3❑	3❑
II (45° - 90°)	4❑	4❑	4❑	4❑	4❑	4❑	4❑	4❑	4❑	4❑
III (90° - 135°)	5❑	5❑	5❑	5❑	5❑	5❑	5❑	5❑	5❑	5❑
IV (>135°)	6❑	6❑	6❑	6❑	6❑	6❑	6❑	6❑	6❑	6❑

Q32. Type of procedure performed

	LEFT HAND					RIGHT HAND				
	Thumb	Index	Middle	Ring	Baby	Baby	Ring	Middle	Index	Thumb
Needle Fasciotomy/aponeurotomy	1❑	1❑	1❑	1❑	1❑	1❑	1❑	1❑	1❑	1❑

Fasciotomy (subcutaneous or open)	2❑	2❑	2❑	2❑	2❑	2❑	2❑	2❑	2❑	2❑
Fasciectomy/Aponeurectomy	3❑	3❑	3❑	3❑	3❑	3❑	3❑	3❑	3❑	3❑
Dermofasciectomy	4❑	4❑	4❑	4❑	4❑	4❑	4❑	4❑	4❑	4❑
Amputation	5❑	5❑	5❑	5❑	5❑	5❑	5❑	5❑	5❑	5❑
Other specify:________________________	6❑	6❑	6❑	6❑	6❑	6❑	6❑	6❑	6❑	6❑

Q33. Duration of the procedure performed: |____| minutes or |____| hours

Q34 Has this patient received any previous **procedure** on the **same** finger or joint(s) from yourself or any other physician?

1❑ Yes *go to Q35.*

2❑ No or not to my knowledge *go to Q38.*

Q35. *If yes in Q34*, what was **the previous** procedure used on the **same** finger/joint

	**LEFT HAND**					**RIGHT HAND**				
	Thumb	Index	Middle	Ring	Baby	Baby	Ring	Middle	Index	Thumb
Needle Fasciotomy/aponeurotomy	1❑	1❑	1❑	1❑	1❑	1	1❑	1❑	1❑	1❑

						❑				
Fasciotomy (subcutaneous or open)	2❑	2❑	2❑	2❑	2❑	2 ❑	2❑	2❑	2❑	2❑
Fasciectomy/Aponeurectomy	3❑	3❑	3❑	3❑	3❑	3 ❑	3❑	3❑	3❑	3❑
Dermofasciectomy	4❑	4❑	4❑	4❑	4❑	4 ❑	4❑	4❑	4❑	4❑
Other specify:______________________	5❑	5❑	5❑	5❑	5❑	5 ❑	5❑	5❑	5❑	5❑

Q36. When did this last previous procedure take place?

|__|__| month (MM) / |__|__|__|__| year (YYYY)

Q37. Who decided upon/performed this previous procedure?

1 ❑ Yourself

2 ❑ GP

3 ❑ Orthopedic Surgeon

4 ❑ Orthopedic surgeon specializing in hand surgery

5 ❑ Plastic surgeon

6 ❑ Plastic Surgeon specializing in hand surgery

7 ❑ Rheumatologist

8 ❑ Other (please specify)_________________

9 ❑Don’t know

PROCEDURE FOLLOW-UP (for the procedure performed between September and December 2008)

Q38. What type of immediate post-operative dressing/splint was applied? (Responses: Light dressing/Bulky bandage/Plaster slab/Thermoplastic splint/Other: specify/None; multiple answers possible)

Q39. *(Except if “none” in Q38)* how long was it used?

|_______| days

❑ Don’t know?

Q39bis. a) Was a Night Splint used? (Responses: Yes/No/Don’t know)

b) (if yes) How long was it used?

|_______| nights

❑ Don't know?

Q40. During the procedure performed between September and December 2008, were there any complications associated with the procedure? (Tick as many as appropriate)

1 ❑ None

2 ❑ Artery injury

3 ❑ Nerve injury

4 ❑ Tendon injury

5 ❑ Volar plate injury

6 ❑ Other. Please specify____________________

Q41. After the procedure performed between September and December 2008, were there any **post-operative** complications **associated with this specific procedure** ? (Tick as many as appropriate)

1 ❑ None

2 ❑ Infection

3 ❑ Hematoma

4 ❑ Complex Regional Pain Syndrome (CRPS) or Reflex Sympathetic Dystrophy or Algodystrophy

5 ❑ Inflammation

6 ❑ Finger required amputation

7 ❑ Abnormal sensitive reactions (Dysesthesia, Paresthesia, Allodynia)

8 ❑ Necrosis

9 ❑ Pain

10 ❑ Carpal tunnel syndrome / Ulnar nerve compression (eg cubital tunnel syndrome)

11 ❑ Wound healing complications / delayed healing

12 ❑ Other (specify):_______________

❑ Don't know?

Q42. *If this patient experienced post operative complications*, did the patient have to be re-admitted in hospital to manage the complications? (Responses: Yes/No/Don't know)

Q43. *If yes in Q42*, how many times has the patient been re-admitted to manage complications of their Dupuytren's contracture procedure?

|___| times

❑ Don't know?

Q44a. How did you assess the effectiveness of the procedure?

1 ❑ By measuring the post-operative flexion or extension

2 ❑ By conducting a table top test

3 ❑ By assessing the patient's functional ability post surgery

4 ❑ Other (please specify)__________________

Q44b. How would you describe the clinical outcome of the procedure perfomed?:

1 ❑ it had a positive outcome

2 ❑ it had no effect on the degree of contracture

3 ❑ it had a negative outcome

Q45. How long did it take to obtain the optimal hand function result following the procedure (taking into account that all patients may not fully regain their optimal hand function)?

|___| months

Q46. What was the optimal result obtained following surgery ? (stage of Dupuytren's Disease of **the operated fingers ;** total finger flexion adding up all the joints affected)

	**LEFT HAND**					**RIGHT HAND**				
	Thumb	Index	Middle	Ring	Baby	Baby	Ring	Middle	Index	Thumb

No nodule nor contracture	1❑	1❑	1❑	1❑	1❑	1❑	1❑	1❑	1❑	1❑
N (Nodules)	2❑	2❑	2❑	2❑	2❑	2❑	2❑	2❑	2❑	2❑
Ia (< 20°)	3❑	3❑	3❑	3❑	3❑	3❑	3❑	3❑	3❑	3❑
Ib (20° - 45°)	4❑	4❑	4❑	4❑	4❑	4❑	4❑	4❑	4❑	4❑
II (45° - 90°)	5❑	5❑	5❑	5❑	5❑	5❑	5❑	5❑	5❑	5❑
III (90° - 135°)	6❑	6❑	6❑	6❑	6❑	6❑	6❑	6❑	6❑	6❑
IV (>135°)	7❑	7❑	7❑	7❑	7❑	7❑	7❑	7❑	7❑	7❑

Q47. Were the hand function limitations expressed by the patient at the diagnosis stage resolved after treatment? (see items in Q20):

	Yes	No	Don't Know
Work activities in general	1❑	2❑	3❑
Leisure activities in general	1❑	2❑	3❑

Washing or grooming	1❑	2❑	3❑
Shaking hands	1❑	2❑	3❑
Placing hand in pocket	1❑	2❑	3❑
Putting on a glove	1❑	2❑	3❑
Difficulty using fingers to grasp objects	1❑	2❑	3❑
Applauding	1❑	2❑	3❑
Using a computer or typing	1❑	2❑	3❑
Problems with recreational or sport activities such as golf or tennis	1❑	2❑	3❑
Baking	1❑	2❑	3❑
Playing a musical instrument	1❑	2❑	3❑
Gardening	1❑	2❑	3❑
Include "Other.1" from Q20 ___	1❑	2❑	3❑
Include "Other.2" from Q20 ___	1❑	2❑	3❑

Q48. Following the procedure, who else managed the patient for the Dupuytren's Disease? (tick all that apply)

1 ❑ No one else but me

2 ❑ GP

3 ❑ Orthopedic Surgeon

4 ❑ Hand surgeon

5 ❑ Plastic surgeon

6 ❑ Rheumatologist

7 ❑ Physiotherapist / occupational therapist

8 ❑ Other (please specify)__________________

Q49. How many clinical visits since the procedure did you see this patient for his/her Dupuytren's contracture follow-up?

|___| times

Q50a. Has there been any other procedure conducted since the procedure for Dupuytren's contracture? (Responses: Yes/No)

Q50b. If Yes, specify if it was:

1 ❑ A recurrence on the same finger/same joints

2 ❑ A procedure on other joints because of disease progression or extension on other joints

3 ❑ A procedure on other joints that was initially planned but had to be delayed

Q50c. if a procedure was conducted on the same finger and same joints:

c1. When did this treatment take place?

|__|__| month (MM) / |__|__|__|__| year (YYYY)

c2. What was the stage of Dupuytren's Disease of **the operated fingers at the time of this new procedure**? (total finger flexion adding up all the joints affected)

	**LEFT HAND**					**RIGHT HAND**				
	Thumb	Index	Middle	Ring	Baby	Baby	Ring	Middle	Index	Thumb
N (Nodules)	1❑	1❑	1❑	1❑	1❑	1❑	1❑	1❑	1❑	1❑
Ia (< 20°)	2❑	2❑	2❑	2❑	2❑	2❑	2❑	2❑	2❑	2❑
Ib (20° - 45°)	3❑	3❑	3❑	3❑	3❑	3❑	3❑	3❑	3❑	3❑
II (45° - 90°)	4❑	4❑	4❑	4❑	4❑	4❑	4❑	4❑	4❑	4❑
III (90° - 135°)	5❑	5❑	5❑	5❑	5❑	5❑	5❑	5❑	5❑	5❑
IV (	6❑	6❑	6❑	6❑	6❑	6❑	6❑	6❑	6❑	6❑

>135°)										

Q50d. if no procedure was conducted on the same finger and same joints:

d1. When did you last reevaluate the operated finger and joints?

|__|__| month (MM) / |__|__|__|__| year (YYYY)

d2. What was the stage of Dupuytren's Disease of **the operated fingers at the time of this last re-evaluation**? (total finger flexion adding up all the joints affected) *(possibly no contracture anymore)*

	LEFT HAND					RIGHT HAND				
	Thumb	Index	Middle	Ring	Baby	Baby	Ring	Middle	Index	Thumb
No contracture nor nodule	1❑	1❑	1❑	1❑	1❑	1❑	1❑	1❑	1❑	1❑
N (Nodules)	2❑	2❑	2❑	2❑	2❑	2❑	2❑	2❑	2❑	2❑
Ia (< 20°)	3❑	3❑	3❑	3❑	3❑	3❑	3❑	3❑	3❑	3❑
Ib (20° - 45°)	4❑	4❑	4❑	4❑	4❑	4❑	4❑	4❑	4❑	4❑
II (45° -	5❑	5❑	5❑	5❑	5❑	5❑	5❑	5❑	5❑	5❑

90°)										
III (90° - 135°)	6❑	6❑	6❑	6❑	6❑	6❑	6❑	6❑	6❑	6❑
IV (>135°)	7❑	7❑	7❑	7❑	7❑	7❑	7❑	7❑	7❑	7❑

FUTURE TREATMENTS PLANNED

Q51. Do you plan to apply a treatment to any hand in the **coming 12 months**?

	**LEFT HAND**					**RIGHT HAND**				
	Thumb	Index	Middle	Ring	Baby	Baby	Ring	Middle	Index	Thumb
No treatment planned / I do not know yet	1❑	1❑	1❑	1❑	1❑	1❑	1❑	1❑	1❑	1❑
Needle Fasciotomy/aponeurotomy	2❑	2❑	2❑	2❑	2❑	2❑	2❑	2❑	2❑	2❑
Fasciotomy (subcutaneous or open)	3❑	3❑	3❑	3❑	3❑	3❑	3❑	3❑	3❑	3❑
Fasciectomy/Aponeurectomy	4❑	4❑	4❑	4❑	4❑	4❑	4❑	4❑	4❑	4❑
Dermofasciectomy	5❑	5❑	5❑	5❑	5❑	5❑	5❑	5❑	5❑	5❑
Steroid Injections	6❑	6❑	6❑	6❑	6❑	6❑	6❑	6❑	6❑	6❑
Amputation	7❑	7❑	7❑	7❑	7❑	7❑	7❑	7❑	7❑	7❑

Radiotherapy	8❑	8❑	8❑	8❑	8❑	8❑	8❑	8❑	8❑	8❑
Other, specify:________	9❑	9❑	9❑	9❑	9❑	9❑	9❑	9❑	9❑	9❑

Q52. When do you plan to carry out this treatment?

0-3 months	1❑	1❑	1❑	1❑	1❑	1❑	1❑	1❑	1❑	1❑
In 3 to 6 months	2❑	2❑	2❑	2❑	2❑	2❑	2❑	2❑	2❑	2❑
In 6 to 12 months	3❑	3❑	3❑	3❑	3❑	3❑	3❑	3❑	3❑	3❑

Q53. Are you planning to refer the patient to another physician or surgeon for treatment of their Dupuytren’s contracture?

1 ❑ Yes (go to Q54)

2 ❑ No (please fill another patient case record form)

Q54. *If yes in Q53*, what physician do you expect to refer this patient to for treatment?

1 ❑ Orthopedic Surgeon

2 ❑ Hand Specialist

3 ❑ Plastic Surgeon

4 ❑ Rheumatologist

5 ❑ Other (please specify)______________________________
